# Consumption Patterns and Willingness to Pay for Sustainable Aquatic Food in China

**DOI:** 10.3390/foods13152435

**Published:** 2024-08-01

**Authors:** Hao Xu, Tianqi Wu, Mausam Budhathoki, Dingxi Safari Fang, Wenbo Zhang, Xin Wang

**Affiliations:** 1 China-ASEAN “The Belt and Road” Joint Laboratory of Mariculture Technology, Shanghai Ocean University, Shanghai 201306, China; haoxu.688@gmail.com (H.X.); tianqiwu12@gmail.com (T.W.); 2 Centre for Research on Environmental Ecology and Fish Nutrition of the Ministry of Agriculture, Shanghai Ocean University, Shanghai 201306, China; 3 Shanghai Engineering Research Center of Aquaculture, Shanghai Ocean University, Shanghai 201306, China; 4 Institute of Aquaculture, University of Stirling, Stirling FK9 4LA, UK; mausam.budhathoki@stir.ac.uk; 5 Department of Food Science, University of Copenhagen, Rolighedsvej 26, 1958 Frederiksberg, Denmark; 6 Emmett Interdisciplinary Program in Environment and Resource, Stanford University, Stanford, CA 94305, USA; 7 Best Aquaculture Practices (BAP), Global Seafood Alliance (GSA), Portsmouth, NH 03801, USA

**Keywords:** seafood, fish, sustainability, consumer behaviour, willingness to pay, food safety, certification, ecolabel, environmental impact, luxury

## Abstract

China, as the world’s largest producer, trader, and consumer of aquatic foods, lacks comprehensive research on consumption patterns and willingness to pay for sustainable aquatic food. This study addressed this gap through an online survey of 3403 participants across Chinese provinces. A majority of consumers (34.7% of the participants) consume aquatic food twice or more per week, mainly from traditional markets (26%). Most prefer fresh or live products (76%), with 42% seeing no difference between farmed and wild options. Consumption is higher among older, affluent, urban, and coastal residents. Crustaceans, especially shrimp, are frequently consumed species, with growing interest in luxury species like salmon and abalone. Taste and quality emerge as the primary factors motivating consumer choices in aquatic food purchases. Food safety is the primary concern, followed by environmental impact. Notably, 92.4% of participants would pay extra for certified products. Factors influencing a higher willingness to pay include higher income, inland residence, price sensitivity, origin consciousness, and concerns about food safety and the environment. The findings highlight that China’s aquatic food industry and consumption can become more sustainable by aligning with consumer preferences for high-quality and diverse aquatic food through both production and import, while also addressing concerns related to food safety and environmental impact. This research provides valuable insights into China’s rapidly transforming aquatic food market landscape, offering implications for industry innovation and the promotion of sustainable consumption patterns.

## 1. Introduction

Aquatic foods, which include any types of food such as fish, shellfish, cephalopods, and aquatic plants derived from both freshwater and marine sources, are vital for food security and nutritional health [1,2]. These foods account for about one-fifth of global animal protein intake and are rich in essential micronutrients like calcium, zinc, iodine, iron, and vitamins A, B, and D [3,4]. Additionally, they are a primary dietary source of omega-3 fatty acids [5]. As the world population grows and dietary patterns evolve, the significance of aquatic foods in ensuring nutritional well-being continues to increase [6,7].

China, as the world’s largest producer and consumer of aquatic foods, has attracted considerable global attention [8,9,10,11]. Spanning over 30 provinces, China’s growing population and economic development have driven demand for aquatic foods across geographical and temporal scales [12,13]. Ongoing societal and economic advancements [13], along with shifts in international trade dynamics [14], have positioned China’s aquatic food consumption at a critical juncture [8]. The diverse range of aquatic foods offers China’s consumers varied choices, resulting in diverse preferences. Unlike the limited variety of terrestrial animal foods, aquatic foods encompass a broad spectrum of species, with over 2500 species currently being consumed. This diversity provides numerous nutritional options, often surpassing meat foods in nutritional value [3].

China, as the world’s largest developing nation, has experienced rapid economic growth. This growth has resulted in an estimated 180 to 200 million Chinese people seeing increased consumer affordability, contributing to China now accounting for one-third of global luxury consumption [15]. Aquatic foods often command higher prices than other protein sources like meat, poultry, eggs, and legumes, frequently positioning them as luxury foods [16]. Despite China’s significant development in aquaculture production, which has produced many traditional species such as carp, they are widely available to consumers [10,17]. However, due to the inconsistency in consumption capacity among Chinese consumers, there has been an increase in the diversity of aquatic food consumption demand [13,16,18,19], leading to a substantial demand for luxury aquatic food species that may even outstrip supply [20,21,22].

China is one of the countries with the fastest urbanization rates in the past few decades [23]. The urban-rural income gap resulting from this rapid urbanization is evident in food consumption patterns [24,25]. Major urban areas in China, particularly in first-tier cities, have emerged as significant markets for luxury aquatic food due to their large population base and the rising income of the middle class [26,27]. Age is also correlated with aquatic food consumption, with younger demographics consuming less aquatic food compared to adults, possibly due to the latter’s relatively higher energy requirements and affordability due to high income [16,18]. Geographic location plays a crucial role in aquatic food consumption patterns; coastal residents consume considerably more aquatic foods than inland residents [28]. Furthermore, urban areas consistently demonstrate higher aquatic food consumption rates than rural regions, regardless of coastal or inland location [29].

The diversity of production methods, sales forms, and purchasing channels for aquatic foods has led to different consumer preferences. Aquatic foods are primarily produced through aquaculture and capture fisheries [30]. In China, more than four-fifths of aquatic food is produced through aquaculture [4,17]. As aquaculture becomes the dominant production method, consumers express concerns regarding its safety and environmental impact, leading to divergent preferences between farmed and wild foods [31,32]. The sales format of aquatic foods is closely related to their species; for example, salmon is typically sold fresh or frozen, tuna is often processed into canned or pre-prepared dishes [33], and carp are generally sold live [34] in China. Regarding purchasing channels, consumers traditionally acquire aquatic foods from traditional wet markets and supermarkets [35]. However, with improving economic conditions and transportation systems, e-commerce has become increasingly popular for aquatic food purchases, particularly among young consumers residing in urban areas [36].

Many factors influence the consumption behavior of aquatic food consumers [37], such as environmental protection [38], food safety [39], animal welfare [40], and so on. Food safety hazards, such as residual antibiotics in aquaculture foods [31], have led many consumers to perceive wild foods as safer than farmed alternatives [16]. Environmental impacts arise from the use of fish oil and fishmeal in aquatic food production and supply chains [10], as well as from aquaculture wastewater [41]. The welfare requirements of aquatic animals, including their sentience, are often inadequately addressed in food systems [40,42]. In terms of product features, consumers focus on factors such as price, quality, taste, purchasing platforms, brands, and packaging [43,44,45]. These aquatic food motivation factors will affect the choice of sustainable aquatic product consumption [46].

Certification and labeling of aquatic foods significantly influence food safety and sustainable consumption [47,48]. The presence of certification labels enhances product value and provides sustainability-related information, reducing consumer ignorance regarding sustainable aquatic foods [49]. Consumers generally exhibit a willingness to pay premium prices for aquatic foods bearing sustainability or organic labels [50]. Elucidating Chinese consumers’ awareness of and support for certified foods could assist producers in better meeting diverse market demands and expectations [51].

When selecting aquatic foods, consumers’ preferences are significantly influenced by their objective and subjective knowledge, socio-demographic background, discriminatory abilities, and attitudes toward aquatic food supply chains [19]. Previous surveys have been conducted in China, but they were either not comprehensive enough or are now outdated and unable to accurately reflect current consumption patterns and preferences. Our study on aquatic food consumption in China improves upon earlier research by utilizing a larger sample size, with data collected from all provinces in China [26,28,52]. Respondents exhibit a more comprehensive range of social characteristics and product preferences [52,53]. The study provides insights into the types of aquatic food commonly consumed by Chinese consumers, as well as luxury varieties [21,54]. Additionally, we have included factors that consumers are concerned about and their willingness towards certification [37,47].

Therefore, our research questions focus on the following aspects:

What are the current consumption patterns, preferences, and habits of Chinese consumers regarding aquatic foods?

How do Chinese consumers’ socio-demographic characteristics relate to their preferred consumption patterns and habits?

Which aquatic foods are most consumed by Chinese consumers, and which luxury foods do they aspire to consume?

What are the important motives and concerns of Chinese consumers when purchasing aquatic foods?

What is the level of awareness and willingness to pay for aquatic food certification among Chinese consumers?

## 2. Materials and Methods

### 2.1. Online Survey and Data Collection

**Figure 1:**
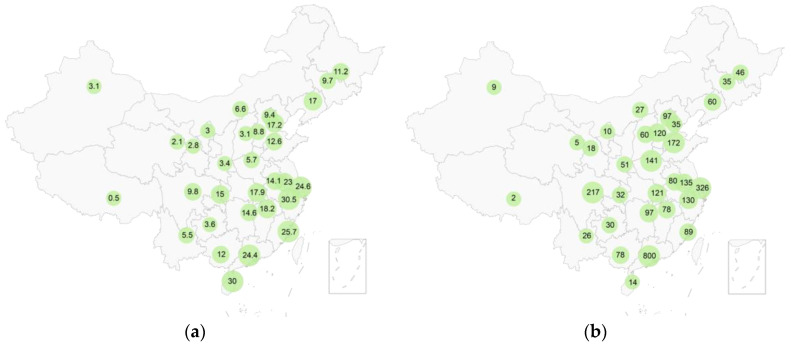
Per capita consumption of aquatic foods in China and distribution of survey participants. (**a**) Per capita consumption of aquatic foods by province in China (kg/year). Data source: National Bureau of Statistics; (**b**) Distribution of survey participants by province.

To overcome geographical constraints and gather data from a diverse, widespread sample, we opted for an online survey approach. This method enabled us to reach respondents across various provinces in China, resulting in a substantial sample size of 3403 participants. Online surveys can introduce sampling bias, underrepresenting groups with limited internet access or digital literacy. However, the benefits of achieving broad geographical coverage outweighed this limitation for our study’s purposes. This study was conducted using Tencent Wenjuan (URL: https://wj.qq.com/), one of the most popular survey platforms in China [55]. The survey questionnaire was distributed on the platform from 8 June to 15 June 2023. Participation was completely voluntary, and informed consent was obtained from all participants. Participants received small monetary compensation for their participation through the Tencent Wenjuan platform. The Ethics Committee of Shanghai Ocean University advised that this study did not require specific approval. This determination was based on the anonymous nature of the research and its adherence to personal privacy protection, consistent with standards applied to previous consumer survey studies [56]. Provincial information for the majority of respondents was obtained through the Tencent Wenjuan platform. However, some participants were not geolocated due to disabled geographic permissions on their devices. The distribution of survey participants across various Chinese provinces is illustrated in Figure 1.

### 2.2. Questionnaire Structure

The questionnaire was developed in the Chinese language and was inspired by previous studies aimed at understanding consumer patterns and willingness to pay [21,28,49,50,52]. The content validity of the questionnaire was determined by two experts in the field of consumer science, while the questionnaire was validated among 30 aquatic consumers. The final questionnaire was prepared based on feedback from the pilot testing and content validity. The questionnaire consists of four main sections with 16 variables in total: (1) sociodemographic characteristics; (2) eating and purchasing habits; (3) perception, preference, and willingness to pay; and finally (4) open-ended questions. Demographic information includes age, income, and residence (urban/rural, inland/coastal). It is worth noting that our survey questionnaire did not include gender as a personal characteristic. This decision was based on previous research findings, which showed no significant difference in aquatic food consumption between males and females [18]. We are more concerned about changes in aquatic food consumption patterns caused by demographic changes, such as income growth, urbanization, and age changes [57]. The second section covers eating frequency, usual place of purchase, preservation form of the product, and production method. The third section covers questions comparing aquatic food and red meat consumption, and questions about respondents’ awareness and willingness to pay for certified aquatic foods. The consumer’s willingness to pay for certified aquatic food was measured by the following item: “How much more are you willing to pay for eco-labelled aquatic food?”; ¥0, ¥0.1 to ¥1, ¥1.1 to ¥3, and ¥3.1 or more.

Open-ended questions primarily focused on the aquatic food most frequently consumed by consumers, the luxury aquatic food they desire to consume, and the motives and concerns they consider when evaluating consumption. For example, one of the open-ended questions was: “Please list 1–3 aquatic foods you consume most frequently”. Species responses were collected and categorized according to commodity species names and broader species categories. In cases where a specific species group was not provided, the food items were assigned to general categories: “Unspecified fish”, “Unspecified crustaceans”, or “Unspecified molluscs” [29]. This classification system ensures that all responses are accounted for, even when detailed species information is unavailable. A five-point Likert scale ranging from 1 “very unimportant” to 5 “very important” was used to assess aquatic food consumption motives and concerns [58].

### 2.3. Consumption Data

Data on the mean consumption of aquatic foods at the provincial level in China for the year 2022 were obtained from the National Bureau of Statistics [59]. Figure 1 illustrates the per capita aquatic food consumption across Chinese provinces. Aquatic foods produced in China predominantly remain within domestic markets [60], and those imported are primarily utilized for processing rather than domestic consumption [9]. Consequently, the preponderance of aquatic foods consumed by Chinese consumers originates from domestic sources. Data on domestic aquatic food production were collected from FAOStatJ software version 4.04 [61], and the proportional distribution of production across species categories of aquatic foods was calculated for 2010 and 2022.

### 2.4. Data Management and Statistical Analysis

Data preparation was conducted using Excel 2022 (Microsoft), and analysis was conducted using SPSS version 28 [62]. Descriptive statistics for consumer socio-demographic characteristics, eating and purchasing habits, perceptions, and preferences were reported using percentages and numbers for categorical variables. Aquatic food choice motives and consumer concerns were presented in a graph as the mean and standard deviation. To visualize some of the data, we used R Studio version 4.4 [63].

Logistic regression is well-suited for analyzing complex relationships in socio-demographic data and consumer behavior, as it does not assume a linear connection between dependent and independent variables. This flexibility allows for more accurate results in consumer analysis research [64]. For this study, a binary logistic regression model was selected because the dependent variable—consumer willingness to pay a higher price—is dichotomous. This model is specifically designed for such binary outcomes, making it the optimal choice for our analysis.

Binary logistic regression was conducted to assess the likelihood of willingness to pay higher prices for certified aquatic food based on their socio-demographic characteristics, aquatic food choice motives, and concerns. The dependent variable was whether consumers were willing to pay higher prices or not, whereas the independent variables were socio-demographic characteristics, aquatic food choice motives, and concerns.

The function of the binary logistic regression model used in this study is represented as follows:

(1) $$ln\left(\frac{P_{i}}{{1-P}_{i}}\right)=\beta_{0}+\beta_{1}X_{i1}+\beta_{2}X_{i2}+\ldots +\beta_{16}X_{i16}+e_{i}$$

where ln denotes the natural logarithm, $\beta_{0}$ is constant, $\beta_{1}$, $\beta_{2}$,… $\beta_{16}$ are vectors of coefficients associated with predictor variables $X_{i1}$, $X_{i2}$,…. $X_{i16}$*,* respectively. $e_{i}$ is an error term. In binary logistic regression, the coefficient calculates a change in log odds of the dependent variable, not the change in the variable itself. So, we also interpreted these coefficients in terms of *odds ratios* with the following exponential function:

(2) $$Odds\;Ratio=e^{\beta_{0}+\beta_{1}X_{i1}+\beta_{2}X_{i2}+\ldots +\beta_{16}X_{i16}+e_{i}}$$

Here, the *odds ratio* is simply the ratio of the probability that the consumers are willing to pay a higher price for certification based on their socio-demographic characteristics, aquatic food choice motives, and concerns. The higher *odd ratios* (*OR* > 1) signify that consumers who belong to specific sociodemographics, prioritize specific factors for buying aquatic food, and are concerned with certain aspects of aquatic food are likelier to pay a higher price for certified aquatic food. Results are presented as odd ratios with associated *p*-values and confidence intervals. Further, ratio and proportion were used to simplify the explanation of the willingness to pay for certified aquatic food. *p*-values less than 0.05 were considered significant and are presented in bold.

## 3. Results

### 3.1. Consumer Characteristics

Table 1 presents the sociodemographic characteristics of survey participants. The largest proportion, 36.61%, was in the 25–34 age group, followed by 29.56% in the under-24 age group. Respondents over 45 years of age accounted for 6.41% of the sample. The primary income brackets were 3000–6000 YUAN and 6000–10,000 YUAN per month, comprising 26.18% and 26.15% of participants, respectively. The lowest income category, below 3000 YUAN per month, included 8.85% of respondents.

Regarding residential distribution, 60.33% of consumers resided in inland areas, while 39.67% lived in coastal regions. Additionally, 45.78% of respondents were from first-tier cities, 26.54% from third- and fourth-tier cities, 19.51% from second-tier cities, and 8.17% from towns and villages. These findings suggest that, while aquatic food consumption is closely associated with coastal areas, inland consumers also represent a significant market. Urban residents, particularly those in first-tier cities, comprise the primary consumer base for aquatic foods.

Figure 1 illustrates the per capita aquatic foods consumption and the number of online survey participants across Chinese provinces. The majority of respondents were from coastal provinces, aligning with the general distribution of aquatic food consumption. As shown in Figure 1a, Guangdong Province and Shanghai Municipality exhibited high per capita aquatic foods consumption rates of 24.4 and 24.6 kg/year, respectively. Correspondingly, Figure 1b indicates that these two regions had the highest number of survey participants, with 800 from Guangdong and 326 from Shanghai. Conversely, western provinces such as Tibet, Qinghai, and Xinjiang demonstrated lower per capita consumption rates of 0.5, 2.1, and 3.1 kg/year, respectively. This lower consumption presumably contributed to fewer survey participants from these regions, with fewer than 10 respondents each.

### 3.2. Eating and Purchasing Habits

Table 2 presents Chinese consumers’ consumption patterns and preferences regarding aquatic foods. Approximately 34.70% of consumers reported consuming aquatic foods twice or more per week, 31.82% consumed them 3–4 times per month, and 24.27% consumed them 1–2 times per month. Only 9.20% of consumers reported consumption less than once per month. Traditional markets and offline supermarkets were identified as the primary purchasing channels, accounting for 26.61% and 25.21% of purchases, respectively. Online e-commerce platforms also played a significant role, representing 15.74% of purchases. Fresh or live foods were the most frequently purchased form (76.61%), followed by chilled (11.70%), prepared foods (7.41%), and frozen (4.29%). 40.17% of consumers prefer wild products, and 42.82% of consumers are willing to accept both farmed and wild aquatic food. Regarding certified foods, 29.21% of consumers reported encountering ecolabel such as BAP when purchasing aquatic foods, while 24.24% had not seen them, and 46.55% were unsure if they had seen them.

### 3.3. Consumption Species

**Figure 2:**
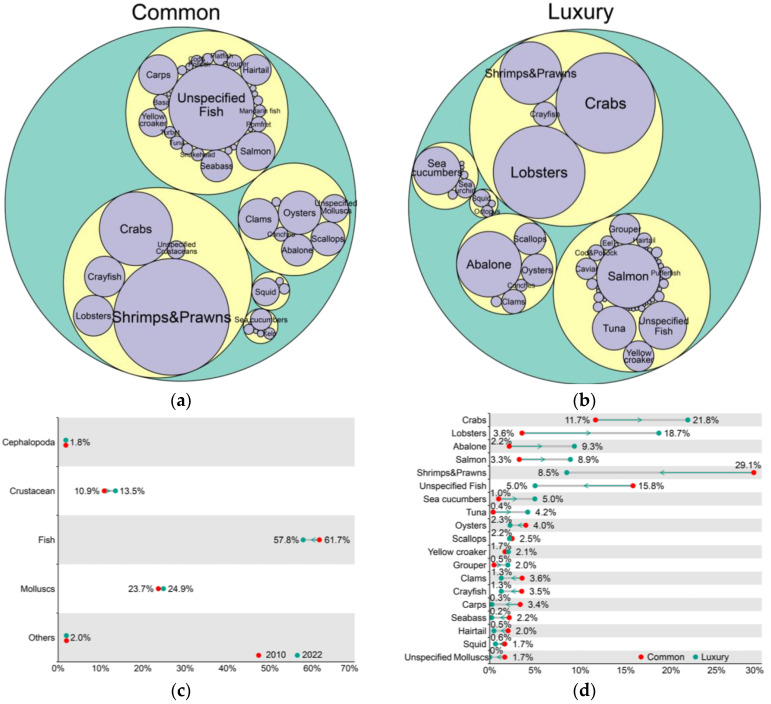
Categories and species of aquatic foods consumed and desired by Chinese consumers. (**a**) Commonly consumed species; (**b**) Desired luxury species; Circle size represents quantity; labels are displayed for aquatic food mentioned more than 20 times; green circles denote all aquatic foods; yellow circles indicate major categories and purple circles represent specific species; (**c**) Changes in production volume of major aquatic food categories in China; (**d**) Percentage changes in commonly consumed species (>1%) and desired luxury species (>1%).

Consumer feedback suggests that crustaceans and fish are the most commonly consumed aquatic food species categories (Figure 2a,b). Crustaceans constitute the highest proportion at 48.58%, with shrimp or prawns being the most frequently consumed species. Fish represent 33.30% of consumption, with cyprinids being the most common after unspecified fish, followed by salmon. Molluscs represent 14.45% of consumption, with clams and oysters being the most frequently consumed species. Cephalopods comprise 2.02% of consumption, primarily squid. Other categories constitute the smallest share, at 1.64% of total consumption. In summary, crustaceans and fish combined represent over 80% of consumption, indicating high consumer demand and acceptance, particularly for crustaceans, which dominate the market.

Regarding desired luxury aquatic foods, consumers also favor crustaceans and fish. Crustaceans rank highest at 50.30%, with crab (king crab, snow crab) and lobster being the most desired. Fish account for 26.87%, with salmon emerging as the most sought-after luxury species. Molluscs represent 15.62%, with abalone being highly popular in the luxury market, followed by oysters and scallops. Other categories constitute 6.18%, predominantly sea cucumber. Cephalopods show the lowest consumer desire at 1.03%. Overall, crustaceans and fish combined account for over 77% of desired consumption, with luxury foods such as abalone, crab, crayfish, and octopus occupying major market shares, reflecting high consumer demand and recognition for these species.

Consumer feedback on frequently consumed species deviates from the actual supply. In China’s aquatic food production (Figure 2c), fish constitute the highest proportion, followed by mollusks, and then crustaceans. The proportions of major categories have changed, with fish production decreasing from 61.7% in 2010 to 57.8% in 2022, while crustaceans and mollusks increased from 10.9% and 23.7% to 13.5% and 24.9%, respectively.

Figure 2d depicts changes in Chinese consumers’ frequently consumed and desired high-end aquatic food species. Notably, crab, lobster, abalone, salmon, and sea cucumber exhibit significant increases in proportion, indicating consumer desire and the gap between aspiration and reality. The substantial decrease in shrimp and prawn proportions implies that frequent consumption has diminished the perceived luxury status of these species.

### 3.4. Consumption Motives and Concerns

**Figure 3:**
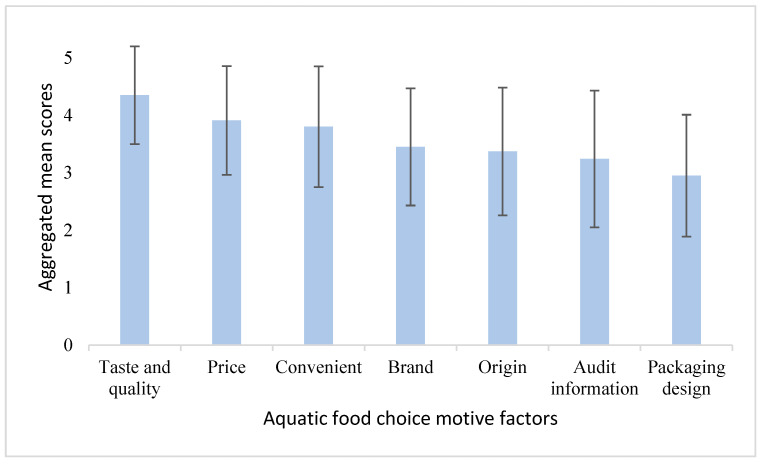
Aggregated mean scores for raking of aquatic food choice motives. Note: Error bar = Standard deviation.

**Figure 4:**
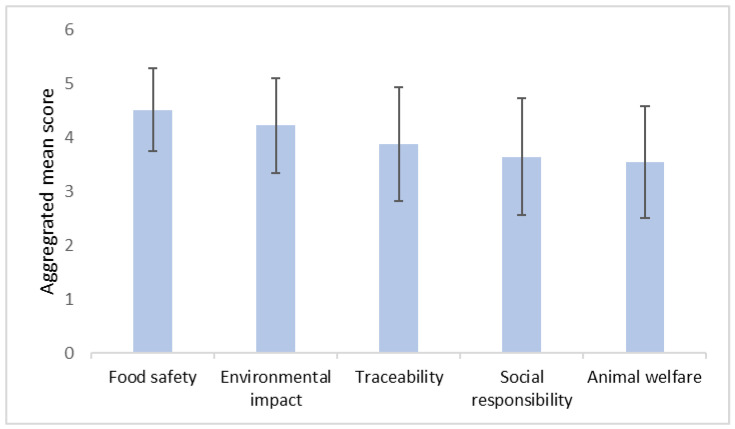
Aggregated mean scores for ranking production environment and processing concern about aquatic food. Note: Error bar = Standard deviation.

Figure 3 and Figure 4 highlight the primary factors both motive and concerns shaping Chinese consumers’ preferences toward aquatic food among Chinese consumers. When making purchase decisions, consumers prioritize taste and quality above all else. Price is the second most crucial factor with convenience following closely. Other considerations, in descending order of importance, are brand, country of origin, inspection information, and packaging design. In terms of production environment and processing concerns, food safety stands out as an important issue. The high aggregated mean score reflects the critical importance Chinese consumers attach to the safety of their aquatic food. Environmental impact ranks as the second most important concern, followed by traceability, social responsibility, and animal welfare.

### 3.5. Perception, Preferences, and Willingness to Pay towards Sustainable Aquatic Food

When comparing the perception between red meat and aquatic foods (Table 3), 53.36% of respondents perceived both as having distinct advantages. Notably, 28.62% considered aquatic foods healthier and planned to increase their consumption, while only 1.73% viewed red meat as the healthier option.

Approximately 55.80% of consumers expressed willingness to pay a premium of 1.1–3 YUAN for foods certified by authoritative third-party auditors, while 7.58% were unwilling to pay any premium for certified eco-labeled aquatic foods. The presence of certification labels positively influenced purchasing intentions for 56.74% of consumers, while 35.91% viewed certification favorably but reported no impact on their purchasing decisions, only 0.41% of consumers expressed aversion to certification labels.

The binary logistic regression analysis revealed that several factors significantly influence consumers’ willingness to pay (WTP) for certified eco-labeled aquatic food (Table 4). Monthly income significantly predicted WTP, with consumers earning above 6000 YUAN showing a higher likelihood of paying a premium (*β* = 0.316, *OR* = 1.372, *p* < 0.001). Geographical location also played a role, as inland residents demonstrated a greater inclination to pay higher prices compared to coastal residents (*β* = 0.208, *OR* = 1.232, *p* = 0.014). Frequent consumers, defined as those purchasing aquatic food more than three times per month, exhibited a higher WTP (*β* = 0.203, *OR* = 1.225, *p* = 0.17), although this effect was not statistically significant at the conventional *p* < 0.05 level. Origin-conscious consumers showed a significantly higher likelihood of paying premium prices (*β* = 0.206, OR = 1.229, *p* = 0.024). In contrast, price-sensitive consumers demonstrated a lower WTP (*β* = −0.460, *OR* = 0.631, *p* < 0.001). Food safety concerns emerged as a strong predictor of WTP (*β* = 0.439, *OR* = 1.552, *p* = 0.002). Similarly, environmental impact concerns positively influenced WTP (*β* = 0.267, *OR* = 1.306, *p* = 0.016). The effects of social responsibility (*β* = 0.180, *OR* = 1.197, *p* = 0.067) and traceability concerns (*β* = 0.181, *OR* = 1.198, *p* = 0.064) approached statistical significance. Factors that did not significantly influence WTP, included age, brand preference, convenience, inspection information, taste and quality, packaging design, and animal welfare concerns.

### 3.6. Consumption Preference Changed with Socioeconomic Characteristics

**Figure 5:**
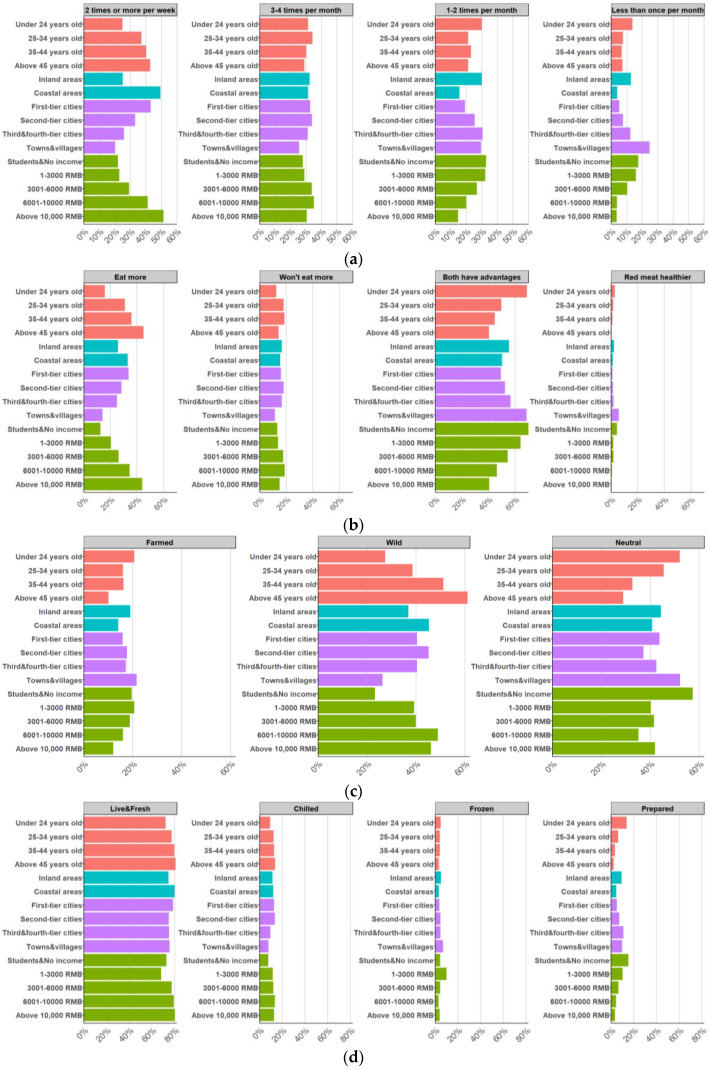
Consumer characteristics and preferences for aquatic foods. (**a**) Frequency of consumption; (**b**) Preference for aquatic foods compared to red meat; (**c**) Preferred production methods; (**d**) Preferred product forms.

Consumers aged 45 and above exhibited the highest frequency of aquatic food consumption, with 42.66% consuming such foods twice or more per week (Figure 5a). Conversely, only 24.85% of consumers under 24 years of age reported similar consumption patterns. A positive correlation was observed between income levels and consumption frequency, with 51.35% of high-income consumers (earning over 10,000 YUAN) consuming aquatic food more than twice weekly. Conversely, lower-income groups exhibited a higher proportion of infrequent consumers (less than once monthly), with 17.56% of students or individuals without income falling into this category, compared to only 3.60% of high-income consumers. Urban residents exhibited higher consumption frequencies than their rural counterparts, with 43.07% of first-tier city dwellers consuming aquatic food twice or more weekly, compared to 20.14% of rural consumers. Furthermore, coastal residents exhibited higher consumption rates than inland residents, with 49.41% and 25.04% consuming aquatic food twice or more weekly, respectively.

Age was identified as a notable determinant, with 44.95% of consumers aged 45 and above considering aquatic foods healthier and expressing intentions to increase consumption, compared to only 15.81% of those under 24 years of age (Figure 5b). Younger consumers predominantly perceived both aquatic foods and red meat as having distinct advantages (68.89% for those under 24 years of age). Income levels also influenced perceptions, with high-income consumers more likely to consider aquatic foods as healthier and express intentions to increase consumption. Among consumers earning over 10,000 YUAN, 44% maintained this perspective, compared to 12.37% of students or those without income. The latter group largely maintained a neutral stance towards both aquatic foods and red meat (69.92%). Urban-rural disparities were evident, with 33.57% of first-tier city consumers recognizing the health benefits of aquatic foods and intending to increase consumption, compared to 14.03% of rural consumers. Geographic location also influenced perceptions, as 33.04% of coastal residents considered aquatic foods as healthier and intended to increase consumption, whereas 50.3% of inland consumers perceived both aquatic foods and red meat as having distinct advantages.

Age emerged as a notable determinant, with 61.01% of consumers aged 45 and above preferring wild foods, compared to only 27.44% of those under 24 years old (Figure 5c). Income levels also influenced preferences, with high-income consumers demonstrating a stronger inclination towards wild foods. Among consumers earning over 10,000 YUAN, 46.1% preferred wild foods, while only 12.01% opted for farmed alternatives. Conversely, lower-income groups showed higher acceptance of farmed foods (19.54%). Urban-rural disparities were evident, with 40.37% of first-tier city consumers favoring wild foods, compared to 26.26% of rural consumers. Geographic location also played a role, as coastal residents exhibited a stronger preference for wild foods (45.26%) over farmed ones (14.07%). In contrast, inland consumers showed a more balanced preference, with 36.82% favoring the wild and 18.95% preferring farmed foods.

This preference was most pronounced among consumers aged 45 and above (80.73%), whereas younger consumers under 24 exhibited a relatively lower preference (72.07%) (Figure 5d). Higher-income consumers exhibited a significantly stronger preference for fresh or live and chilled aquatic foods compared to lower-income groups. As income levels increased, the preference for live foods also increased, whereas lower-income consumers tended to prefer frozen and prepared options. Among students or consumers without income, 72.07% preferred fresh or live foods, 4.87% selected frozen foods, and 13.72% preferred prepared items. Conversely, among consumers earning over 10,000 YUAN, 80.03% preferred fresh or live foods, 3.75% selected frozen foods, and only 3.45% opted for prepared options. Urban consumers exhibited a slightly higher preference for fresh or live foods (78.56%) compared to rural consumers (75.54%). Nonetheless, rural consumers exhibited higher acceptance of frozen (6.83%) and prepared (9.71%) foods compared to urban consumers (3.59% and 5.07%, respectively). Regional differences were also observed, with coastal consumers exhibiting a stronger preference for fresh or live (79.93%) and chilled (12.15%) foods compared to inland consumers (74.43%, and 11.40%, respectively). Conversely, inland consumers exhibited a higher acceptance of frozen (4.97%) and prepared (9.21%) foods compared to coastal consumers (3.26% and 4.67%, respectively).

## 4. Discussion

### 4.1. Diversified Aquatic Food Demands along Economic Development

Aquatic food, with its diverse species and consumption patterns, is intricately connected to the broader food system [65]. These products serve as a vital nutritional source, offering a healthier alternative to red meat while exerting less environmental impact, positioning them as exemplars of sustainable future food choices [3,66]. Notably, over 25% of Chinese consumers view aquatic food as a beneficial substitute for red meat and intend to increase their intake. This trend not only indicates growing consumption but also underscores the potential for sustainable consumption within China’s aquatic product market and overall food ecosystem.

Rapid economic development has increased affordability, driving demand for these luxury foods [13,15,22]. Leveraging its dominant position in aquaculture, China has witnessed a diversification in commonly consumed aquatic species. However, a discrepancy exists between reported consumption frequencies and actual supply. For instance, while carp have the largest supply and are reported as the most consumed fish by consumers, they are not the most consumed aquatic food species overall. Similar phenomena occur in the United States, where shrimp consumption frequency is not proportional to its supply [29]. The apparent discrepancy between consumption frequency and supply of aquatic food may be attributed to several factors. Crustaceans, for instance, might be consumed more frequently than fish, albeit in smaller portions. Additionally, premium crustaceans like shrimp and lobster often feature prominently in festive occasions and gatherings, occupying a special place in consumers’ minds. Chinese consumer preferences have, to an extent, shaped production trends, as evidenced by the gradual shift from fish to crustacean and mollusk production, reflecting a growing appetite for diverse, high-quality aquatic food.

For Chinese consumers, quality, taste, and price are the most important motivational factors for choosing aquatic food. The popularity of luxury aquatic food species such as crab (king crab, snow crab), lobster, salmon, abalone, and sea cucumber demonstrate Chinese consumers’ pursuit of healthy, high-priced, and unique imported aquatic foods [22,54]. These high-value products not only fulfil nutritional needs but also serve as status symbols, particularly among consumers in first-tier cities and the burgeoning middle class [21,26,27]. This demographic’s willingness to pay premium prices for novel or rare aquatic food could potentially steer China’s aquatic industry towards higher quality and value-added development.

Looking forward, Chinese consumers are expected to increase their consumption of imported shrimp and non-native species like salmon and lobster [8,9,54]. This trend signifies more than just evolving taste preferences; it heralds a broader transformation in China’s food consumption landscape. Such changes are likely to have far-reaching implications for both domestic production and international trade in the aquatic product sector.

### 4.2. Evolving Consumption Patterns with Social Development

China’s food system is experiencing a rapid transformation, primarily driven by social factors such as urbanization and economic growth [67]. This shift is reflected in changing aquatic product consumption patterns, which are influenced by demographic changes, particularly rising incomes, urbanization, and an aging population [57].

Food culture plays a complex role in shaping food choices, acting as both a cause and effect. Increased population mobility, improved transportation, and the spread of diverse dietary cultures can lead to changes in eating habits [68]. Although the number of consumers and consumption volume is still higher in coastal provinces, some provinces in the central regions also have a certain number of consumers and consumption volume. This indicates that aquatic food consumption is expanding from coastal provinces to inland central regions. As these areas develop economically and consumer habits evolve, the market is expected to expand further. However, lower-income groups are less likely to consume aquatic food, possibly due to price considerations, highlighting the need for targeted strategies to address price sensitivity [16].

Inland consumers have a higher acceptance of farmed products compared to coastal consumers, which may be influenced by the aquaculture culture resulting from the rapid growth of aquaculture production in inland China [17]. More than half of consumers prefer farmed products or have no preference, but wild products remain popular among certain groups, including the elderly, high-income groups, urban residents, and coastal consumers. This preference may stem from perceptions of superior taste, quality, and nutritional value associated with wild products, as well as lingering concerns about farmed product quality and aquaculture practices [69,70]. The perception of wild products as more “natural” and “pure” underscores the need for the aquaculture industry to improve transparency and sustainability to gain consumer trust. Enhancing the quality of farmed products and increasing public awareness of modern aquaculture practices are crucial for the industry’s continued development [71,72,73].

These trends highlight the complex interplay between socioeconomic factors, cultural perceptions, and consumer preferences in shaping China’s aquatic product market. As the market evolves, addressing these multifaceted challenges and opportunities will be key to ensuring sustainable industry growth and meeting the diverse needs of different regions and populations.

### 4.3. Willingness to Pay Sustainable Aquatic Food

Our study revealed food safety as the paramount concern for consumers and an important factor in influencing willingness to pay for sustainable aquatic food. This finding corroborates previous research, underscoring the significant impact of food safety concerns on Chinese consumers’ aquatic product consumption behavior [37,74]. The concern likely stems from recent food safety incidents and increased public health awareness [75]. Notably, environmental impact and traceability closely follow as key concerns, suggesting that consumers are taking a more holistic approach when evaluating aquatic food, considering not only personal benefits but also broader social and environmental impacts.

Compared to the previous one-third of Chinese consumers who are unwilling to pay extra for certified food [49], our research indicates that over 90% of Chinese consumers are willing to pay a premium for sustainable aquatic product certification, reflecting a positive market perception of sustainable aquatic food. Food safety concerns have emerged as a strong predictor of willingness to pay, with environmental concerns also significantly impacting willingness to pay. This trend highlights consumers’ growing awareness of these factors and their readiness to support related products.

In China, the prevailing belief is that market participants are not primarily responsible for environmental governance, which has hindered the implementation of market-based initiatives [22]. However, consumers are increasingly willing to pay a premium for eco-certified sustainable aquatic products due to concerns about food safety and environmental impact. This shift provides valuable insights for policymakers and producers in promoting sustainable consumption. Policymakers can leverage the demonstrated willingness to pay for certified sustainable products by implementing incentives for environmentally friendly practices. Producers and retailers can address consumer concerns about food safety and environmental impact by investing in transparent supply chains and adopting blockchain technology for traceability. Such measures not only meet consumer demands but also potentially command premium prices in the market. By highlighting rigorous safety protocols and eco-friendly practices, manufacturers and retailers can set their products apart in the marketplace, creating valuable opportunities for differentiation. However, effectively communicating these attributes and ensuring claim credibility pose significant challenges. The industry must navigate these issues carefully to maintain consumer trust and support the growing demand for sustainable and safe aquatic food.

Certification labels emphasizing green or eco-sustainable practices and including information on food safety and environmental impact should be promoted, given their high social acceptance in China [50]. To meet evolving consumer expectations, a credible certification system covering the entire production process is urgently needed [75]. Such a system would not only enhance consumer confidence in food safety but also stimulate demand for high-quality aquatic food.

As the market evolves, addressing these multifaceted consumer concerns will be crucial for the sustainable growth of China’s aquatic food industry. By aligning production practices with consumer values and effectively communicating these efforts through a credible certification system, the industry can foster trust, drive innovation, and contribute to more sustainable long-term consumption patterns.

## 5. Conclusions

Chinese consumers’ aquatic food consumption patterns are evolving in tandem with socioeconomic changes. Economic development has fuelled demand for premium aquatic foods, driving a trend towards species diversification. Consumption patterns exhibit variation across demographic groups. Older, affluent, urban, and coastal consumers demonstrate a stronger preference for fresh, live, and wild products. This highlights the intricate interplay between socioeconomic factors, cultural perceptions, and consumer preferences in shaping aquatic food market dynamics. As the world’s largest seafood market, China’s seafood consumption patterns have been influenced by urbanization, economic growth, and demographic changes. This not only provides valuable insights for other rapidly developing countries but also helps predict dietary shifts and reveal global demand trends.

Food safety has emerged as the primary concern among consumers, closely followed by environmental impact considerations. Notably, the proportion of consumers willing to pay a premium for sustainably certified aquatic food has surged beyond 90%, presenting opportunities for product differentiation based on safety and environmental sustainability credentials. To address evolving consumer expectations, China’s aquatic food consumption market requires the development of robust certification systems encompassing the entire production process. By effectively communicating safety standards and environmental practices, aligning with consumer values, and implementing transparent certification, the aquatic food industry can cultivate trust, drive innovation, and promote sustainable consumption patterns in China’s rapidly transforming market landscape.

## Figures and Tables

**Table 1:** Sociodemographic characteristics of respondents: N = 3403.

Sociodemographic Characteristics	Categories	% (n)
Age	Under 24 years old	29.56 (1006)
	25–34 years old	36.61 (1246)
	35–44 years old	27.42 (933)
	Above 45 years old	6.41 (218)
Monthly income (YUAN)	No Income	19.25 (655)
	3000 YUAN or below	8.85 (301)
	3001–6000 YUAN	26.18 (891)
	6001–10,000 YUAN	26.15 (890)
	Above 10,001 YUAN	19.57 (666)
Region (Inland/Coastal)	Inland area	60.33 (2053)
	Coastal area	39.67 (1350)
Residence (Urban/Rural)	First-tier city	45.78 (1558)
	Second-tier city	19.51 (664)
	Third/Fourth-tier city	26.54 (903)
	Village/Town	8.17 (278)

**Table 2:** Eating and purchasing habits of the participants.

Variables	Categories	%(n)
Frequency of aquatic food consumption	Twice a week or more	34.70 (1181)
	3–4 times a month	31.82 (1083)
	1–2 times a month	24.27 (826)
	Less than once a month	9.20 (313)
Usual place of purchase	Small community stores or community group purchases	10.10 (820)
	E-commerce	15.74 (1278)
	Traditional markets (fish market)	26.61 (2160)
	Supermarkets	25.21 (2046)
	Family cooking	11.26 (914)
	Restaurants/takeout	11.08 (899)
Preservation form	Fresh or live	76.61 (2607)
	Chilled	11.70 (398)
	Frozen	4.29 (146)
	Prepared	7.41 (252)
Production method	Farmed	17.01 (579)
	Wild	40.17 (1367)
	Both	42.82 (1457)
Have you ever noticed certification label while purchasing aquatic food, such as Best Aquaculture Practice (BAP)?	Yes	29.21 (994)
	Maybe	46.55 (1584)
	No	24.24 (825)

**Table 3:** Chinese consumers’ perception and preferences towards aquatic food.

Variables	Categories	%(n)
Aquatic foods are a better source of nutrition than red meat	Yes, I will eat more in the future	28.62 (974)
	Yes, but I will not eat more in the future	16.28 (554)
	Both have their advantages; I will not change my diet ratio	53.36 (1816)
	No, red meat is healthier	1.73 (59)
Certification in aquatic food	Think it is good, but it will not affect my purchase decision	35.91 (1222)
	More willing to buy	56.74 (1931)
	No impact	6.94 (236)
	Dislike it	0.41 (14)
Willing to pay extra for certified eco-labeled aquatic food	0 YUAN	7.58 (258)
	0.1 to 1 YUAN	21.30 (725)
	1.1 to 3 YUAN	55.80 (1899)
	3.1 YUAN or more	15.31 (521)

**Table 4:** Factors influencing higher willingness to pay for certified eco-labeled aquatic food.

Factors	*β*	Standard Error	*Odd Ratio*	Confidence Interval	*p*-Value
Age (35 and above = 1) **Income (¥6000 and above = 1)** **Region (Inland = 1)**	−0.005	0.087	0.995	0.839–1.181	0.956
	**0.316**	**0.087**	**1.372**	**1.157–1.628**	**<0.001**
	**0.208**	**0.085**	**1.232**	**1.043–1.455**	**0.014**
Aquatic food consumption (>3 times a month) **Motives**	0.203	0.085	1.225	1.036–1.448	0.17
				
**Price**	**−0.460**	**0.094**	**0.631**	**0.525–0.759**	**<0.001**
Brand	0.024	0.088	0.785	1.024–0.863	0.785
Convenient	−1.06	0.090	0.899	0.754–1.072	0.236
Inspection information	0.152	0.093	1.164	0.970–1.397	0.103
**Origin**	**0.206**	**0.091**	**1.229**	**1.028–1.470**	**0.024**
Taste and quality	0.160	0.123	1.174	0.923–1.493	0.190
Packaging design	0.036	0.098	1.037	0.856–1.257	0.711
**Concerns**					
**Food safety**	**0.439**	**0.140**	**1.552**	**1.180–2.041**	**0.002**
**Environmental impact**	**0.267**	**0.111**	**1.306**	**1.052–1.622**	**0.016**
Social responsibility	0.180	0.098	1.197	0.988–1.452	0.067
Animal welfare	0.091	0.099	1.095	0.901–1.330	0.361
Traceability	0.181	0.098	1.198	0.989–1.452	0.064
Constant	−0.472	0.214	0.624		

Note: In the model, Hosmer and Lemeshow Test (Chi-square = 14.785, df = 8, *p* < 0.063), and Nagelkerke R Square = 0.071; the bold numbers represented a significantly higher likelihood of willingness to pay extra for each one-unit increase in the corresponding factor.

## Data Availability

The original contributions presented in the study are included in the article, further inquiries can be directed to the corresponding authors.
